# Microbiome in Rheumatoid Arthritis and Celiac Disease: A Friend or Foe

**DOI:** 10.7759/cureus.15543

**Published:** 2021-06-09

**Authors:** Kawther Elsouri, Vania Arboleda, Samantha Heiser, Marc M Kesselman, Michelle Demory Beckler

**Affiliations:** 1 Medicine, Nova Southeastern University Dr. Kiran C. Patel College of Osteopathic Medicine, Fort Lauderdale, USA; 2 Medicine, Nova Southeastern University Dr. Kiran C. Patel College of Allopathic Medicine, Fort Lauderdale, USA; 3 Rheumatology, Nova Southeastern University Dr. Kiran C. Patel College of Osteopathic Medicine, Davie, USA; 4 Microbiology and Immunology, Nova Southeastern University Dr. Kiran C. Patel College of Allopathic Medicine, Fort Lauderdale, USA

**Keywords:** oral microbiome, celiac disease, gut microbiome, probiotics, rheumatoid arthritis, gut-joint axis, autoimmune diseases

## Abstract

Rheumatoid arthritis (RA) and celiac disease (CD) are both autoimmune diseases with increasing global prevalence. These two diseases have been connected based on similar HLA mutations, serological markers, rheumatological, and gastrointestinal manifestations. In this review, we discuss the role of the oral and gut microbiome in the development and progression of RA and CD. Here, we highlight similar microbial dysbiosis and how these alterations in composition can lead to worsening disease severity in both CD and RA. Additionally, we analyze the role of probiotics in regulating the microbiome and improving symptoms associated with RA and CD.

## Introduction and background

Rheumatoid arthritis (RA) is a chronic systemic autoimmune disease that can manifest clinically as symmetrical joint damage, limited range of motion, and swelling [1]. RA primarily affects the lining of the synovial joints, leading to progressive disability, socioeconomic burdens, and possibly premature death. Poorly controlled or severe cases have a higher risk for extra-articular manifestations: cardiovascular illness, keratitis, pulmonary granulomas/rheumatoid nodules, pericarditis/pleuritis, small vessel vasculitis, and other non-specific symptoms [1]. Within four weeks of gastrointestinal or genitourinary bacterial infections or viral infections, acute or chronic forms of arthritis can arise, which can mimic seropositive RA [2].

There are two major subtypes of RA depending on the presence or absence of anti-citrullinated protein antibodies (ACPAs) [1]. Both subtypes present with joint pain, swelling, and limited range of motion. However, seropositive RA patients, where serum ACPA is present, tend to have more severe manifestations and phenotypes, including higher levels of pain, inflammation, and bone destruction than seronegative RA patients. ACPAs, present in approximately 67% of patients, can be found long before the onset of the joint symptoms [1]. The genetic risk factor associated with ACPA-positive RA is found in the human leukocyte antigen (HLA)-DR gene. The two alleles HLA-DR1 and HLA-DR4 have “shared epitopes” (SEs) [3], and are considered primary risk factors for RA outcomes due to their role in the production of ACPAs [4]. In addition to genetic risk factors, mucosal sites exposed to a high load of bacterial antigens may represent the initial site of autoimmune generation [4]. Emerging data have implicated the microbiome as an extra-articular trigger of RA pathogenesis [4,5].

Following a proper diagnosis, the treatment options for RA include long-term disease-modifying antirheumatic drugs (DMARDs, including biologic agents) and intermittent short courses of corticosteroids, which aim to maintain quality of life [6]. The problem with RA treatments is the severity of the known adverse effects of DMARDs which include liver and GI effects, drug-induced lupus erythematosus, *Clostridium difficile* colitis, and infertility [6].

Celiac disease (CD) is an autoimmune disease whereby its pathogenesis is triggered by the consumption of gluten and gluten components, including wheat, barley, and rye [7]. Clinical manifestations of CD can include villous atrophy of the small intestine, malnutrition, vitamin deficiency, osteoporosis, anemia, chronic diarrhea, cancer, fertility problems, and psychiatric and behavior abnormalities [7]. Similar to RA, microbial and viral infections have been associated with CD development in genetically predisposed patients [7].

CD is diagnosed by villous atrophy on duodenal biopsy and can also include the presence of serum antibodies targeting tissue transglutaminase (tTg) and immunogenic gluten proteins/peptide sequences [8]. In patients with CD, tTg cleaves gluten and produces glutenins and gliadins (33-mer peptide fragment, found in wheat, rye, and barley) which act as highly immunogenic epitopes that have a linked affinity for the protein products of HLA DR3-DQ2 and/or DR4-DQ8 alleles [9]. Moreover, greater than 99% of individuals with CD have HLA DR3-DQ2 and/or DR4-DQ8 alleles [7]. While anti-tTg antibodies are used as a diagnostic tool, the pathogenic mechanism is thought to be T cell-mediated. Patients with active CD tend to exhibit similar levels of CRP and pro-inflammatory cytokines as RA patients due to inflammation to intestinal damage.

Preservation of the intestinal villi is greatly improved with early diagnosis of CD, however difficulty in determining the correct diagnosis limits early detection due to a growing percentage of clinical manifestations being extraintestinal without any gastrointestinal symptoms at all [9]. Currently, treatment plans center around a gluten-free diet (GFD) to control the immune responses observed in CD. However, a diet modification is not effective for all CD patients due to continued, but inadvertent ingestion of gluten, commonly from gluten contamination of products presumed to be gluten-free [10].

## Review

**Figure 1:**
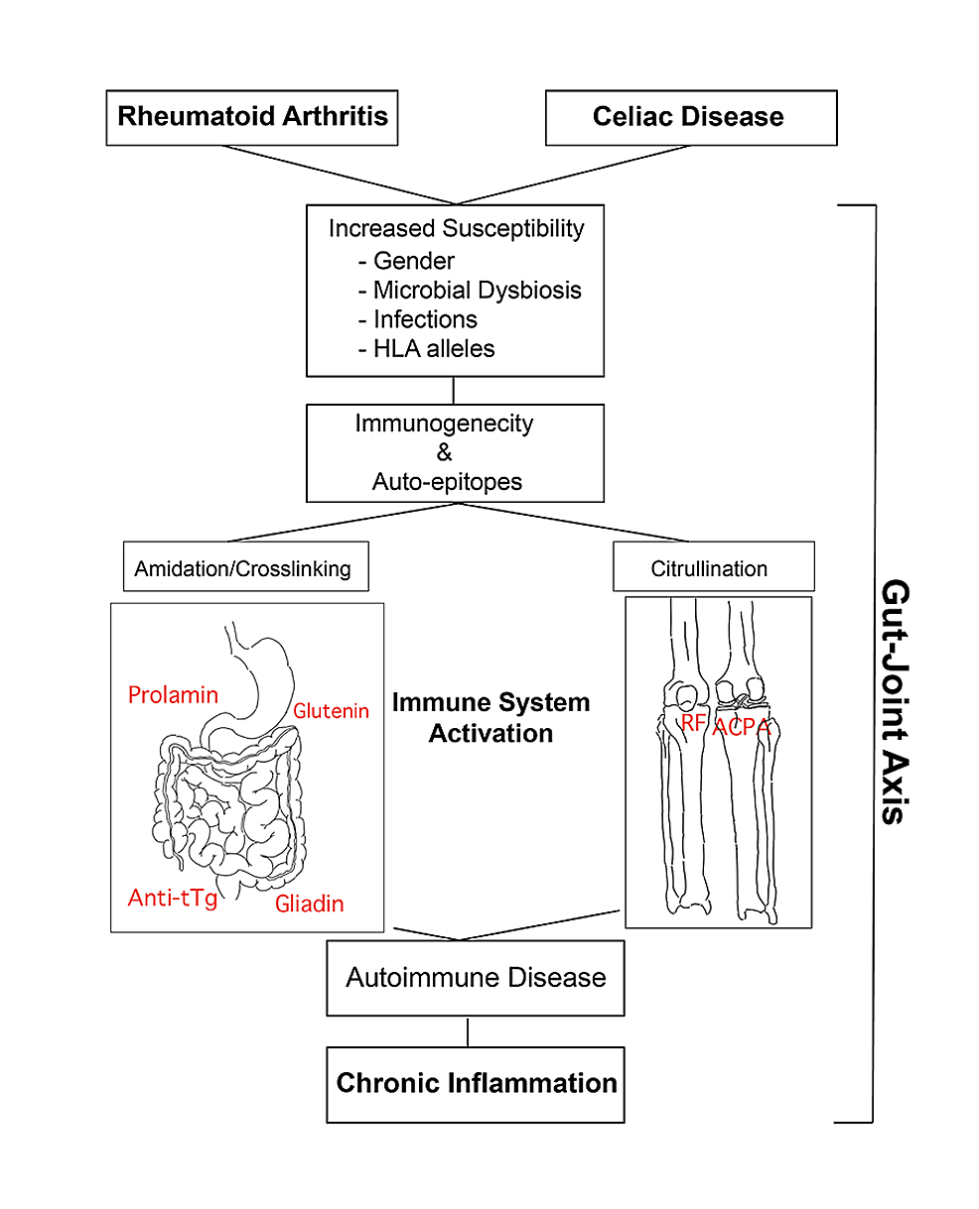
Diagram depiction of the gut-joint axis. HLA: human leukocyte antigen; anti-tTg: anti-tissue transglutaminase antibodies; ACPA: anti-citrullinated protein antibodies; RF: rheumatoid factor

RA and CD share multiple autoimmune pathogenic features and clinical manifestations. The presence of anti-gluten antibodies is more prevalent in RA patients than controls [10], and a similar increase in the presence of tTg auto-antibodies was found in RA [11]. The incidence of CD has increased significantly over the last six decades and an analogous trend can be observed for rheumatic diseases in females and aging populations [7]. Many studies have suggested a gut-joint axis, whereby inflammation in the gut mucosa precedes joint manifestations, up to years in advance, suggesting underlying immune mechanisms and the gastrointestinal environment can influence the onset and progression of joint disease (Figure 1) [7]. A casual link between the onset and progression of RA and CD has been linked. Both have similar clinical presentations, diagnostic features, epidemiological trends, and CD involves chronic autoimmune inflammation of the GI tract (Table 1). To this end, we hypothesize that CD and RA are linked. A major factor that draws a bridge between the two is the gut-joint axis, whereby events occurring in the gut can impact the joints. We sought to understand how dysbiosis of the gut and oral microbiomes as components of the gut-joint axis link CD and RA. Below, we identify specific strains and species involved in the microbiome dysbiosis seen in RA and CD patients and their role in the development and progression of both autoimmune diseases. Additionally, we identify how probiotics may play a role in decreasing disease severity.

**Table 1:** Similarities and differences between patients with RA and CD. Source: Refs. [1-3,7,8,12-16]. ACPA: anti-citrullinated protein antibodies; RF: rheumatoid factor; HLA: human leukocyte antigen; tTg: tissue transglutaminase; EBV: Epstein-Barr virus; CMV: cytomegalovirus; HBV: hepatitis B virus; HCV: hepatitis C virus; RA: rheumatoid arthritis; CD: celiac disease

	Celiac disease	Rheumatoid arthritis
Prevalence	3.3 million in the USA as of 2020	1.36 million in the USA as of 2014
Female:Male ratio	3:1	3:1
Genes associated	HLA-DQ2 and HLA-DQ	HLA-DR4 and HLA-DRB1
Biomarkers	Gliadin, glutenin, prolamin, anti- tTg antibodies	RF and ACPA
Target/associated organs	Target: intestines. Associated: blood, joints/bones, skin, teeth, joints, brain, heart, reproductive organs.	Target: joints/bones. Associated: Intestines, skin, eyes, lungs, heart and vasculature, liver, kidneys, blood, teeth.
Associated triggers	Type 1 diabetes mellitus, autoimmune thyroid disease, Addison’s disease, alopecia, formula-fed	Smoking, obesity, alcohol consumption
Infections associated with the onset	Enterovirus, EBV, CMV, HBV, and rotavirus, bacterial microbes: Bacteroides species, Campylobacter jejuni, Pneumococcus, Mycobacterium tuberculosis, and Helicobacter pylori	Salmonella, Shigella, Campylobacter, Yersinia, and Chlamydia trachomatis, HIV, EBV, Parvovirus, HBV, HCV, Mycoplasma, Proteus Mirabilis

Role of oral and gut microbiomes in RA

The oral cavity harbors over 700 species of bacteria that contribute to the health and physiological status of the oral cavity [17]. The oral microbiome is extremely dynamic due to its constant relationship with the external environment (eating, oxygen availability, communicating, defending against infection, etc.) [18].

Initially, it was thought that it was the humoral immune response to *Porphyromonas gingivalis*, a prominent oral pathogen linked to gingivitis and periodontal disease (PD), which was involved in the onset of RA [19]. Peptidyl-arginine deiminase (PAD) is produced by *P. gingivalis* and can citrullinated antigens/proteins in patients with RA. In RA, citrullination is a physiological process in many tissues and can lead to immune response [20]. Given that PAD can citrullinated antigens, a link was loosely established that increased levels of PAD producing *P. gingivalis* in the oral cavity play a part in activating T cells, which in turn, will provide antigen-specific help to B cells to generate the ACPAs in RA patients [21].

*Aggregatibacter actinomycetemcomitans* and *Anaeroglobus geminatus* were later added to the list of microbes associated with increased disease severity in RA. *A. actinomycetemcomitans* is involved in hypercitrullination in host neutrophils, inducing an immune reaction in RA patients [21], and *A. geminatus*, was correlated with ACPA/rheumatoid factor (RF) presence in RA patients with PD [22].

Another organism, capable of producing large amounts of citrulline, *Cryptobacterium curtum*, emerged as a robust discriminant of the oral microbiome in individuals with RA without PD [23]. Further sequencing determined that dental and salivary samples of patients at high risk for developing RA showed enrichment of *Lactobacillus salivarius*, *Atopobium* spp., and *C. curtum* [24]. *L. salivarius* is prominent in very active cases of RA and was positively correlated with increasing immunoglobulin G (IgG), C-reactive protein (CRP), and erythrocyte sedimentation rate (ESR) levels [25]. Together, these studies showed the importance of oral microbial species in the pathogenesis of RA.

The variations in the gut microbiome in RA patients have also been implicated in RA pathogenesis [5]. The healthy gut microbiome is characterized by three major phyla: *Firmicutes*, *Bacteroidetes*, and *Actinobacteria* [26]. This microbiome serves as a functional expansion of host genomes and is estimated to harbor 50-100 folds more genes than the host [27]. These extra genes add various types of enzymes which are not encoded by the host and play a critical role in facilitating and regulating host metabolism and physiology [28]. In healthy adults, fragments of normal intestinal bacteria are found in circulating blood leukocytes in the spleen. In patients with inflammatory arthritis, similar degradation products of bacterial cell walls and nucleic acids were detected in the inflamed joints [29]. In individuals at risk for RA, the arthritogenic bacterial antigens would pass from the intestines to the joints [30]. This suggests that individuals with genetic susceptibility for RA are prone to harbor intestinal microbiota containing specifically arthritogenic bacterial species or stains leading to aberrant immune responses and chronic inflammation [31].

*Prevotella copri* (Pc) was discovered in new-onset, untreated RA (NORA) patients, as an intestinal microbe correlated with disease activity [32]. Increases in Pc abundance correlated with a reduction in *Bacteroides* and a loss of beneficial microbes in NORA patients [27]. The prevalence of Pc in chronic RA (CRA) patients being treated and exhibiting reduced disease activity, was similar to healthy subjects [33]. One possibility is Pc fails to thrive when there is less inflammation due to a lack of terminal electron acceptors [33]. Despite the links between the intestinal microbiome and RA development, it remains unclear whether these states of oral and gut dysbiosis represent a causal-etiological factor or a secondary effect of local and systemic inflammation.

Role of oral and gut microbiome in CD

The microbiome’s role in CD has been studied extensively due to gluten’s effect on the intestinal membrane [33]. However, little is known regarding the association between dysbiosis and gluten-specific T-cell response relevance in CD [34]. It has been shown that members of the phylum *Proteobacteria* are more abundant and those of the *Firmicutes* and *Actinobacteria* phyla are less abundant in active CD than in GFD patients or controls [34].

Caminero et al. found that bacterial colonization produced distinct gluten-degradation in the mouse small intestine [33]. *Pseudomonas aeruginosa*, an opportunistic pathogen from CD patients, exhibited elastase activity which allows producing of shorter gluten peptides [33]. These peptides often highly immunogenic effectively translocated the mouse intestinal barrier [33]. However, mice incubated with *P. aeruginosa* and *Lactobacillus* spp. showed reduced major immunogenic peptides, suggesting that immunogenic peptides generated by *P. aeruginosa* can be degraded to non-immunogenic peptides in the presence of *Lactobacillus* spp. [33].

In patients with CD and patients with refractory CD (RCD, CD unresponsive/resistant to at least 12 months of treatment with GFD), levels of these bacteria fluctuated [35]. Significant differences between patients with CD and RCD groups in *Bacteroidetes* (CD>RCD), *Actinobacteria* (CD<RCD), and *Fusobacteria* (CD>RCD) were found. The oral microbiome of patients with RCD was significantly different from the one in patients with CD. As CD progresses to RCD, oral microbial colonization patterns change [35]. These changes offer new opportunities for screening, diagnosis, and potential evaluation of the risks of a CD patient transitioning to RCD [35]. Nonetheless, additional research is required to confirm this presumption.

Probiotics as treatment

The Food and Agriculture Organization (FAO)/World Health Organization (WHO), defines a probiotic as a “live microorganism, which when administered in adequate amount confers a health benefit on the host" [36]. The benefits of probiotics on host gut health can be manifested through (i) production of inhibitory substances against pathogens, (ii) blockage of adhesion sites, (iii) competition for nutrients, (iv) degradation of toxin receptors, and (v) regulation of immunity [37]. Over the past five years, new research has shed a light on novel probiotic strains that could be useful for the treatment of RA and CD [38-45].

Probiotics and rheumatoid arthritis 

Since 2015, several novel probiotics have been shown to modulate RA disease severity by altering gut microbe composition in mouse/rat models and human subjects [38-41]. In human subjects with RA, supplementation of *Lactobacillus acidophilus*, *Lactobacillus​​​​​​​ casei*, and *Bifidobacterium bifidum* for eight weeks exhibited an improvement in disease activity score, a significant decrease in serum insulin, and high sensitivity C- reactive protein (hsCRP) levels [39]. The patients showed statistically significant improvement of total and low-density lipoprotein (LDL) cholesterol levels. *L. casei* was shown to lower pro-inflammatory cytokines interleukin-12 (IL-12) and tumor necrosis factor-α (TNF-α) levels with reduced swollen joints after treatment [39]. Intervention with *Bifidobacterium animalis* subspecies (subsp.) *lactis* GCL2505 (BlaG) resulted in reduced severity of diseased joints [40]. Treatment with BlaG has been documented to increase levels of *Lactobacillus* and *Bifidobacterium*, which were positively correlated to the production of protective short-chain fatty acids (SCFAs) [40].

A study using a rat model of arthritis showed a reduction of TNF-α levels with a decrease in disease activity after oral treatment with *Bacillus coagulans* and inulin [41]. The reduction in TNF-α was observed to be similar to the anti-inflammatory effects of indomethacin (NSAID) [41]. *Prevotella histicola* isolated from the human gut showed a reduced incidence of disease with delayed onset in HLA-DQ8 RA induced mice [40]. The treatment has reduced cellular and humoral immunity by lowering autoantibodies and decreasing antigen-specific T-cell proliferation [40]. The decrease in pro-inflammatory cytokines and increase in regulatory cells in the gut led to an increase in tight junction proteins and maintenance of gut epithelial integrity [40]. This study suggests *P. histicola* would be a useful probiotic treatment for RA, especially since the strain is endogenous to the human gut [40]. This possibility would allow for fewer side effects, and the capability to treat patients with targeted antibiotics if side effects do arise [40].

Probiotics and celiac disease

A concoction of *Streptococcus thermophilus, Lactobacillus plantarum, L. acidophilus, L. casei, L. delbrueckii *subsp. *bulgaricus, Bifidobacterium breve, B. longum, B. infantis, L. salivarus spp.* subsp. *salicinius*, known as VSL#3, is the treatment for irritable bowel syndrome (IBS), and ileal pouch [42]. In 2006, Angelis et al. showed how VSL #3 could be useful in treating celiac sprue (CS). The epitopes involved in stimulating an excessive immune response in CS contain a large amount of proline (Pro) residues [46]. As an amino acid, Pro is unique due to its cyclic structure. This conformation establishes many restrictions regarding the structural aspects of peptides and proteins, resulting in increased resistance to hydrolysis [46]. The mass spectrometry showed an almost complete deterioration of gliadins during long-time fermentation of wheat flour by VSL#3 in mouse models [43]. However, VSL#3 was unable to hydrolyze gliadins when each of its component strains were tested separately, this implies that no single probiotic strain in VSL #3 contained all the required peptidases needed to break down gliadins [43].

Several strains alleviate symptoms in patients with active CD: *Bifidobacterium infantis* Natren Life Strain super strain (NLS-SS) [43], *Bifidobacterium breve* strains (BR03 and B632) [44,45], and a cocktail of five strains of lactic acid bacteria and *Bifidobacteria*: *L. casei* LMG 101/37 P-17504, *L. plantarum* CECT 4528, *B. animalis* subsp. *lactis* Bi1 LMGP-17502, *Bifidobacterium breve* Bbr8 LMG P-17501, and *Bifidobacterium breve* Bl10 LMG P-17500 [47]. Pinto-Sanchez et al. tested the impact of *Bifidobacterium infantis* NLS-SS by evaluating Paneth cells, macrophage counts, and human-defensin 5 (HD5) expression in duodenal biopsies of CD patients on a GFD [43]. Their results revealed that patients treated with NLS-SS experienced a decline in HD5 expression and Paneth cell counts [48]. Klemenak et al. explored the action of two* Bifidobacterium breve* strains (BR03 and B632) on serum IL-10 and TNF-α levels in 49 children with CD on GFD, showing lower levels of TNF-α after daily use for three months [45]. Primec et al. carried out a double-blind placebo-controlled study enrolling 40 CD and 16 healthy children [44]. In another study, children with CD were randomized to receive either a placebo or a cocktail of two *Bifidobacterium breve* strains BR03 (DSM 16604) and B632 (DSM 24706) for three months [49]. This probiotic combination was able to alter the production of acetic acid and total short-chain fatty acids and ultimately played a role in stimulating the restoration of the microbiome [44].

## Conclusions

The microbiome is an essential immune modulator, and its dysbiosis is thought to result in a negative impact on health. The research has shed light on a new role of the microbiome in RA and CD, and the intricate relationship between its make-up and the presentation of clinical symptoms. While many crucial issues remain to be identified, some aspects are now evident: (a) patients with RA and CD have alterations in the composition of both their oral and gut microbiome, (b) these alterations are associated with disease activity, and (c) the use of probiotics (in adjunct with disease-centered treatment) have shown to be effective in improving symptom severity by changing the microbial composition of the oral or gut microbiome. Together, these studies strongly indicate an association between dysbiosis of the gut and oral microbiomes that negatively influence the gut-joint axis. These studies support our anecdotal clinical encounters suggesting RA patients with CD have increased rheumatic disease severity (data not shown). Future studies on the pathological role of the microbial strains implicated in CD and RA and the impact of probiotics in reducing their levels are needed to better understand this link.
